# How to demonstrate drug‐induced Type 1 Brugada pattern electrocardiogram

**DOI:** 10.1002/joa3.13170

**Published:** 2024-10-21

**Authors:** Naoya Kataoka, Teruhiko Imamura

**Affiliations:** ^1^ Second Department of Internal Medicine University of Toyama Toyama Japan

To editor:

Data on drug‐induced Type 1 Brugada patterns remain limited. The authors' study examined the clinical characteristics and implications of drug‐induced Type 1 Brugada patterns,^1^ raising several concerns.

During the follow‐up period, 10% of patients exhibited a spontaneous Type 1 pattern,^1^ suggesting that Brugada syndrome may be a progressive disease. Recently, multiple syndromes, including Brugada syndrome, J‐wave syndrome, and idiopathic ventricular fibrillation, have been postulated to represent the same underlying condition: subepicardial cardiomyopathy.^2^ This condition is characterized by an arrhythmogenic substrate located in the epicardium with fibrosis and the progressive nature of Brugada syndrome lends support to this concept. Another critical concern is the identification of patients in whom a spontaneous Type 1 pattern develops. This cohort may demonstrate a higher prevalence of pathogenic variants and delayed enhancement on cardiac magnetic resonance imaging. The prognostic significance of these findings remains a topic of interest.

Due to the small number of events observed in patients with drug‐induced Type 1 patterns, the authors did not advocate for routine sodium channel blocker challenge tests.^1^ However, the underlying mechanisms responsible for these findings remain unclear. The sodium channel blocker challenge test is typically performed to identify arrhythmogenic substrates, which are the therapeutic targets of catheter ablation in symptomatic Brugada syndrome patients.^3^ Should we reconsider the necessity of this drug challenge test to ensure adequate catheter ablation?

The authors' study did not address the presence of early repolarization,^1^ which is often seen in patients with Brugada syndrome and constitutes a risk factor for arrhythmic events.^4^ How many patients had concomitant early repolarization? The response to ajmaline in J‐wave may vary depending on the presence of arrhythmic events or the specific location of early repolarization, an aspect that warrants further investigation.

## CONFLICT OF INTEREST STATEMENT

Authors declare no conflict of interests for this article.
